# Supplementary material for: Characterization of the Genomic Landscape in Cervical Cancer by Next Generation Sequencing
Source: Genes (Basel). 2022 Jan 31;13(2):287. doi: 10.3390/genes13020287 (PMC8871541; doi:10.3390/genes13020287)
Supplement: Supplementary file 1 [file genes-13-00287-s001.zip › genes-1527502-supplementary.pdf]

GCAT TACG GCAT genes

MDPI

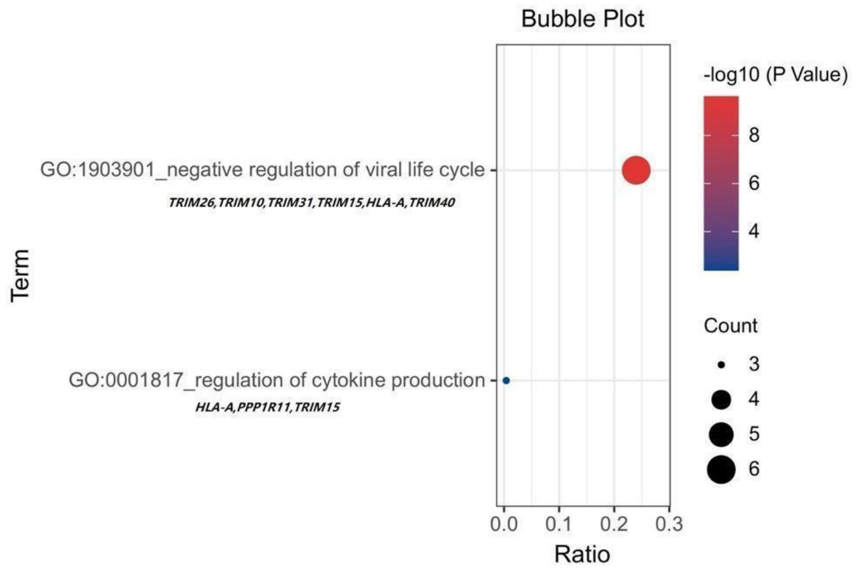

Figure S1. Bubble chart to show the significantly enriched KEGG pathways of the hub genes.

Table S1. Detailed clinical characteristics of the patients in this study.

No.	Age	Tumor Size	T Stage	N	M	Pathologic Diagnosis	Stage	Biomarker	HPV
1	33	0.5 cm	2a1	0	0	Squamous carcinoma	IIA1	Undetected	16, 51
2	34	1 × 0.9 × 0.9 cm	1b1	1	0	Squamous carcinoma	IIIC1p	Undetected	High-risk
3	37	5.5 × 3 × 2 cm	2b2	1	0	Squamous carcinoma	IIIC1	SCCA	16
4	38	3 × 2 cm	2a1	1	0	Squamous carcinoma	IIIC1	Undetected	16
5	38	Undetected	1a1	0	0	Squamous carcinoma	IA1	Undetected	16, 58
6	41	2.5 × 1.5 cm	1b2	0	0	Squamous carcinoma	IB2	Undetected	16

7	41	Undetected	3c1	1	0	Adenocarcinoma	IIIC1	Undetected	16, 18
8	43	5 × 3 cm	1b2	0	0	Squamous carcinoma	IB3	Undefined	High-risk
9	44	4.5 × 3 cm	3C	1	0	Squamous carcinoma	IIIC1	Undetected	16
10	45	6 × 5 cm	2a2	1	0	Undifferentiated carcinoma	IIIC2p	Undetected	negative
11	46	5 × 3.5 cm	1b	1	0	Adenosquamous carcinoma	IIIC1	CA199	18
12	47	5.0 × 4.0 cm	2a	0	0	Adenosquamous carcinoma	IIA2	CA125	Undefined
13	48	Undetected	1a2	0	0	Squamous carcinoma	IB1	Undetected	16
14	49	2.5×2×1.5 cm	2b	0	0	Adenosquamous carcinoma	IIB	Undetected	16
15	49	6 × 4 cm	2b	1	0	Squamous carcinoma	IIIC1	SCCA	16
16	49	2 × 1.5 × 1 cm	2a	0	0	Squamous carcinoma	IIA1	Undetected	Undefined
17	51	2.5 × 2.5 cm	3a	1	0	Endometrioid serous carcinoma	IIIC1	HE4	16
18	51	5.5 × 3.5 cm	2a1	1	0	Squamous carcinoma	IIIC1p	SCCA	16
19	51	3 × 3 × 1.5cm	2a1	1	0	Squamous carcinoma	IIIC1	Undetected	Undefined

20	52	2.8 × 1.8 × 0.8cm	1b	0	0	Squamous carcinoma	IB2	SCCA	16
21	52	6 × 4.5 cm	1b2	1	0	Adenosquamous carcinoma	IIIC1p	Undetected	16
22	52	5cm × 4 cm	1b3	0	0	Squamous carcinoma	IB3	SCCA	Undefined
23	53	3 × 2 × 1.8 cm	1b	0	0	Squamous carcinoma	IB2	CA125	16
24	53	5.0 × 3.0 cm	1b2	0	0	Squamous carcinoma	IIA2	Undefined	Undefined
25	54	5.4 × 4.5 × 1.8 cm	2a2	1	0	Squamous carcinoma	IIIc1p	Undetected	16
26	54	Undetected	1a1	0	0	Squamous carcinoma	IA1	Undetected	33
27	55	Undetected	1a1	0	0	Squamous carcinoma	IB1	Undetected	Undefined
28	57	3 × 1.5 × 0.3 cm	2a	0	0	Squmous carcinoma	IIA1	Undetected	18
29	60	4 × 3 × 1.3 cm	2a2	0	0	Squamous carcinoma	IIA2	Undetected	16
30	62	3 × 2.5 × 2 cm	1b2	0	0	Adenocarcinoma	IB2	CA125	16
31	67	4.6 × 4 cm	2b	1	0	Squamous carcinoma	IIIC1p	Undetected	Undefined
32	77	7 × 4 × 2 cm	2b	X	0	Squamous carcinoma	IIB	CA125,CA199,CEA,SCCA	16

Table S2. Probable oncogenic somatic variations identified in the study and labeled in the ONCOGENIC database.

Gene	Variant_Classification	HGVS.c	HGVS.p	Sample Count	Origin
FBXW7	Nonsense_Mutation	c.832C>T	p.Arg278Ter	1	Somatic
APC	Nonsense_Mutation	c.6629C>G	p.Ser2210Ter	1	Somatic
ARID1B	Frame_Shift_Ins	c.5587_5588insAACTTACT	p.Gly1863fs	1	Somatic
ATR	Frame_Shift_Del	c.4731_4759delTACACAGACTGTGTTCTCCATGCTTGACC	p.Ser1577fs	1	Somatic
ATR	Frame_Shift_Del	c.4761_4764delTCTC	p.His1587fs	1	Somatic
BAP1	Frame_Shift_Del	c.1833_1834delGA	p.Lys612fs	1	Somatic
CIC	Frame_Shift_Del	c.7508delC	p.Pro2503fs	1	Somatic
DAXX	Frame_Shift_Ins	c.1149_1150insCACAGGATCTGATAGTGCAGGGTCAACGCCTACGTGGGAA	p.Asp384fs	1	Somatic
DAXX	Frame_Shift_Ins	c.1153_1154insT	p.Lys385fs	1	Somatic
DNMT3A	Nonsense_Mutation	c.745C>T	p.Gln249Ter	1	Somatic
EP300	Nonsense_Mutation	c.5449C>T	p.Gln1817Ter	1	Somatic
ERBB3	Missense_Mutation	c.1166C>T	p.Thr389Ile	1	Somatic
FAM175A	Nonsense_Mutation	c.205C>T	p.Gln69 Ter	1	Somatic
FAM175A	Splice_Site	c.283-1G>C		1	Somatic
FAT1	Nonsense_Mutation	c.3286C>T	p.Arg1096 Ter	1	Somatic
HLA-A	Nonsense_Mutation	c.757G>T	p.Glu253Ter	1	Somatic
KIT	Missense_Mutation	c.2089C>T	p.His697Tyr	1	Somatic
KMT2C	Nonsense_Mutation	c.14743G>T	p.Glu4915 Ter	1	Somatic
KMT2D	Nonsense_Mutation	c.7333C>T	p.Gln2445 Ter	1	Somatic
KMT2D	Nonsense_Mutation	c.5317C>T	p.Gln1773 Ter	1	Somatic
KMT2D	Nonsense_Mutation	c.14873C>G	p.Ser4958 Ter	1	Somatic
MLH1	Nonsense_Mutation	c.1438G>T	p.Glu480Ter	1	Somatic
NCOR1	Frame_Shift_Ins	c.110_111insCCTCATTACCT	p.Glu37fs	1	Somatic
NF2	Splice_Site	c.115-1G>C		1	Somatic
PALB2	Nonsense_Mutation	c.3477G>A	p.Trp1159Ter	1	Somatic
PTEN	Nonsense_Mutation	c.469G>T	p.Glu15Ter	1	Somatic
PTEN	Nonsense_Mutation	c.640C>T	p.Gln214Ter	1	Somatic
SOX9	Frame_Shift_Ins	c.1138dupC	p.His380fs	1	Somatic
SPEN	Nonsense_Mutation	c.4345G>T	p.Glu1449Ter	1	Somatic
STK11	Nonsense_Mutation	c.193G>T	p.Glu65Ter	1	Somatic
TP53	Frame_Shift_Ins	c.868_869insTGTTAAAAAAAAAAAAAAA	p.Arg290fs	1	Somatic
TP53	Missense_Mutation	c.775G>T	p.Asp259Tyr	1	Somatic
TP53	Missense_Mutation	c.839G>C	p.Arg280Thr	1	Somatic
TP53	Nonsense_Mutation	c.867_868insTTTTCATAGGGGAACTGTAAAATATTTATACAGCTAGCAGCA	p.Leu289_Arg290insPheSerTerGlyAsnCysLysIlePheIleGlnLeuAlaAla	1	Somatic

Table S3. Demographic and baseline characteristics of the study population stratified by FIGO stage.

Variable	Early Stage (FIGO I/II, *n* = 18)	Advanced Stage (FIGO III, *n* = 14)
Age, years		
Median (range)	50.5(33–77)	47.5(34–67)
Histologic type		
Squamous carcinoma	15	9
Adenosquamous carcinoma	2	2
Adenocarcinoma	1	1
Endometrioid serous carcinoma	0	1
Undifferentiated carcinoma	0	1
Biomarkers		
CA125 +	4	0
CA199 +	1	1
SCCA +	3	3
HE4 +	0	1
Undetected	10	9
Undefined	2	0
HPV infection		
HPV 16	8	8
HPV 18	1	2
HPV 33	1	0
HPV 51	1	0
HPV 58	1	0
High risk	1	1
Negative	0	1
Undefined	5	2
